# Cell Organisation in the Colonic Crypt: A Theoretical Comparison of the Pedigree and Niche Concepts

**DOI:** 10.1371/journal.pone.0073204

**Published:** 2013-09-12

**Authors:** Richard C. van der Wath, Bruce S. Gardiner, Antony W. Burgess, David W. Smith

**Affiliations:** 1 School of Computer Science and Software Engineering, University of Western Australia, Perth, Western Australia, Australia; 2 Ludwig Institute for Cancer Research, Royal Melbourne Hospital, Melbourne, Victoria, Australia; 3 The Walter and Eliza Hall Institute of Medical Research, Melbourne, Victoria, Australia; Michigan State University, United States of America

## Abstract

The intestinal mucosa is a monolayer of rapidly self-renewing epithelial cells which is not only responsible for absorption of water and nutrients into the bloodstream but also acts as a protective barrier against harmful microbes entering the body. New functional epithelial cells are produced from stem cells, and their proliferating progeny. These stem cells are found within millions of crypts (tubular pits) spaced along the intestinal tract. The entire intestinal epithelium is replaced every 2–3 days in mice (3–5 days in humans) and hence cell production, differentiation, migration and turnover need to be tightly regulated. Malfunctions in this regulation are strongly linked to inflammatory bowel diseases and to the formation of adenomas and ultimately cancerous tumours. Despite a great deal of biological experimentation and observation, precisely how colonic crypts are regulated to produce mature colonocytes remains unclear. To assist in understanding how cell organisation in crypts is achieved, two very different conceptual models of cell behaviour are developed here, referred to as the ‘pedigree’ and the ‘niche’ models. The pedigree model proposes that crypt cells are largely preprogrammed and receive minimal prompting from the environment as they move through a routine of cell differentiation and proliferation to become mature colonocytes. The niche model proposes that crypt cells are primarily influenced by the local microenvironments along the crypt, and that predetermined cell behaviour plays a negligible role in their development. In this paper we present a computational model of colonic crypts in the mouse, which enables a comparison of the quality and controllability of mature coloncyte production by crypts operating under these two contrasting conceptual models of crypt regulation.

## Introduction

**Figure 1:**
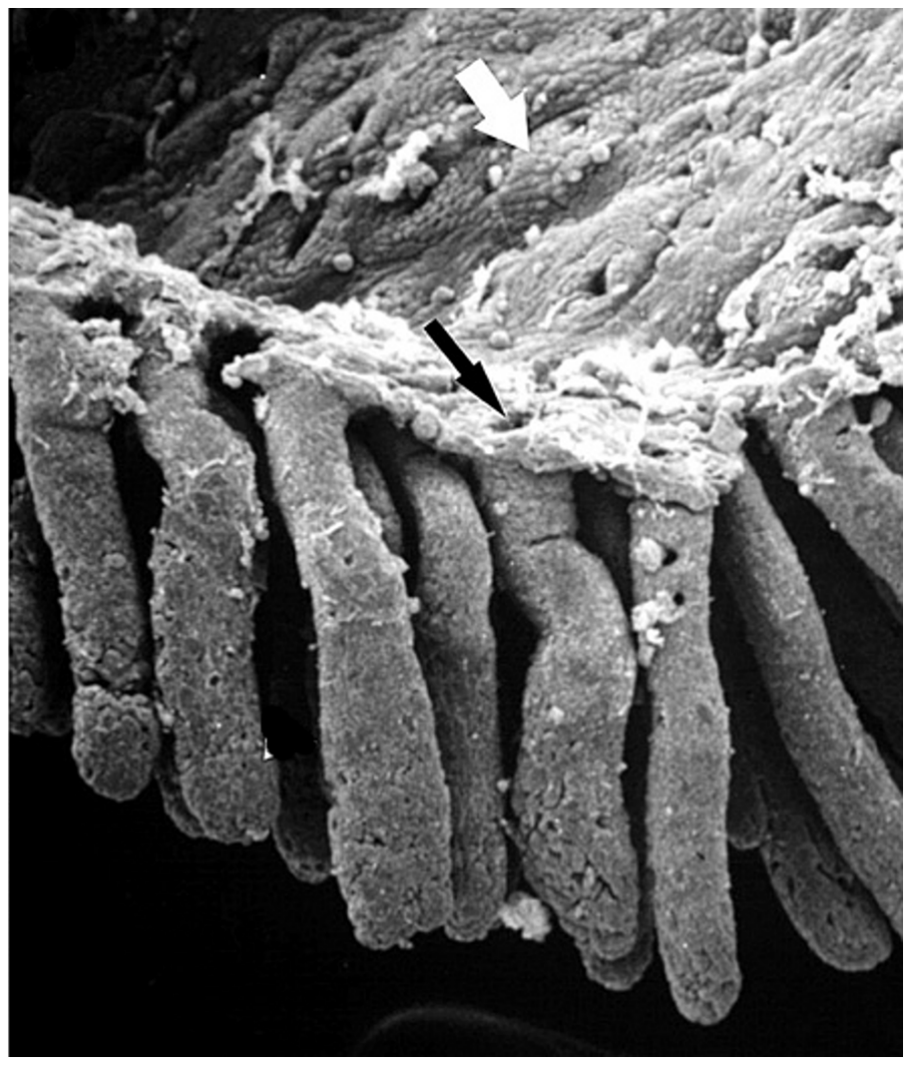
Colonic crypt of the mouse. A scanning electron micrograph of colonic crypt finger-like protrusions in the mouse (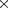
120 magnification). In contrast to the small intestine the lumenal surface of the colon is flat with no no villi (white arrow). The black arrow indicates the orifice of the crypt lumen. (reproduced with permission from [68]).

Many tissues, such as skin and blood, undergo constant regeneration. This self-renewal is accomplished by millions of cells that divide and differentiate to replenish lost functional cells, or to repair the tissue following injury. The self-renewal process evolved into a tightly regulated system and evidence has been found that it includes mechanisms such as asymmetric chromosome segregation in stem cells [1] or dormant stem cell populations which can be reversibly activated upon injury [2]–[4], i.e. presumably when a sudden increase in new cells is required. One of the fastest self-renewal processes found in mammals occurs in the intestinal epithelium, which is replaced every 2–3 days in mice and 3–5 days in humans. The intestinal epithelium exists in a challenging chemical and mechanical environment [5]. This single layer of cells is responsible for both absorption of water and nutrients as well as forming a protective cell-sheet that prevents harmful substances freely entering the lamina propria. The functional epithelial cells that perform these tasks are not themselves proliferating, but are instead the progeny of highly proliferative immature cells found in the small pits (the so-called crypts of Leberkühn) lining the intestinal tract (Figure 1). Details about the distribution of proliferative cells along the length of the crypt, as well as their proliferation rates, have been inferred from labelling-index (LI) studies [6]–[9]. LI data in the crypt is a measure of the mitotic activity along the length of the crypt, defined as the number of cells in the S phase of the cell cycle at each vertical position divided by the total cells in that position. The LI studies revealed a distinct ordering along the crypt among proliferative cells at the crypt base and mature cells at the crypt orifice, an observation confirmed by more recent Ki67 immunostaining (a marker for cell proliferation) [10], [11]. The Ki67 immunostains further suggest that there exists a fairly sharp boundary between the proliferative and mature regions along the crypt and that proliferative cells primarily occupy the lower third of the crypt.

**Figure 2:**
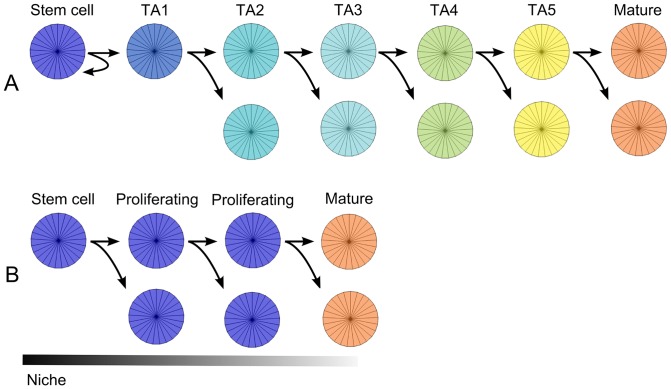
The Pedigree and Niche concept. A: The Pedigree Concept. Key characteristics for this model of self-renewal are a preprogrammed differentiation hierarchy and asymmetric division of stem cells. The differentiation hierarchy and colour coding used by our model are also shown. TA denotes transit-amplifying cells, and the number denotes the cell generation for the TA cell lineage. B: The Niche concept. Maturity and proliferation are determined by the environment. Here we use the same colour for stem cells and other proliferating cells. Note that all cell divisions are symmetric.

Much of the most recent biological research has been focused on finding markers for intestinal stem cells within the proliferative compartment of the crypt [12]. Evidence suggests that stem cells reside at [13] or near the bottom [14] of the crypts, and that their direct progeny (so-called transit amplifying (TA) cells) proliferate rapidly and mature as they migrate along the crypt wall [15]. The cells' differentiation and maturation mechanisms need to be regulated to ensure the production of mature epithelial cells at a suitable rate and quality so as to maintain the integrity of the intestine's epithelial lining. Indeed malfunction in these regulatory processes are strongly linked to the formation of adenomas and ultimately invasive tumours [16], [17]. Unfortunately consensus has not been reached on the precise mechanisms that control self-renewal, differentiation, and proliferation in the crypt. The theories to date can be broadly classified into two schools of thought: the pedigree concept [18]–[22] and the niche concept [15], [23] (Figure 2). The precise meanings attached to these concepts varies in the biological literature, depending on the purpose, and hence it is important to clarify the definitions we employ in this paper.

### Pedigree concept

The pedigree model of stem cell organisation in the crypt imposes a hierarchical heredity on cell types [18], [19], [24] (Figure 2A). The cells at the top of the hierarchy are deemed to be putative stem cells and are assumed to be immortal. Hence all stem cell divisions are ‘asymmetric’, with one daughter cell remaining as a stem cell and the other daughter cell differentiating into a TA cell after mitosis. These assumptions are strongly supported by evidence of asymmetric chromosome segregation [25]–[29] (also known as the ‘immortal strand hypothesis’ [1]). TA-cells then divide symmetrically through several rounds of divisions (at a faster rate than stem cells) and each new generation progressively differentiates to finally reach a mature cell state. Central to the pedigree concept is the notion that the differentiation state (or type) of a cell after each division is predetermined and hence so too is the number of TA-cell generations required for a cell to reach maturity. However, in principle cells in the pedigree model are not entirely unresponsive to environmental cues. Cells could for example, still adapt their resting cell cycle times and attachment affinities in response to a dynamic environment. Apart from the number of TA-cell generations, the other variables specific to this model are the number of stem cells and rates of division of the stem cells and TA-cells.

### Niche concept

The niche model proposes that stemness and differentiation states are not an inherent property of cells but are rather determined by signals received from the local microenvironment [15]. The term ‘niche’ refers to the microenvironment which harbours stem cells. In the case of the colon this niche is typically assumed to be near or at the bottom of the crypt [30]. The conditions and signalling in the niche are such that any cell that enters this space adopts stem cell properties. The microenvironmental conditions change along the crypt (Figure 2B) which causes cells that leave the niche to progressively differentiate and to mature. This process continues as a cell moves along the crypt until the daughter cells have become irreversibly mature. In the niche model all cell divisions are thus symmetric but it is only the daughter cells that remain in the niche that remain stem cells. The observations of monoclonal conversion of a crypt [23], [31]–[33] provides strong evidence in favour of symmetric cell division as the dominant stem cell division mechanism.

In this paper we present a computational framework of the colonic crypt in the mouse that is capable of simulating both the pedigree and niche concepts. We have implemented the two models in order to do a theoretical comparison of crypts regulated by the pedigree and niche concepts, with the aim of assisting biologists in interpreting experimental results on crypt cell organisation and regulation. The pedigree and niche models as we have implemented them represent two extremes. In all probability the reality of crypt behaviour lies somewhere in between the two models. Hence our aim is not to promote either model as being ‘better’ than the other, but simply to compare which assumptions are elevated by each model so as to inform future biological experiments aimed at elucidating the basis of crypt regulation.

Our computational framework represents individual cells as semi-autonomous agents that interact with each other and with their local environment (see the Methods section). Our model can hence be classified as a single cell-based (also called agent-based) lattice-free model of the crypt [34]. Previous mathematical models of the crypt have either assumed a pedigree or niche regime [21], [35]–[38], and except for [38] there is no previous theoretical study to directly compare the two paradigms. Meineke [21] introduced the first lattice-free model of the crypt using Voronoi tessellation to model cell boundaries. Cell organisation in the Meineke model [21] is based on the pedigree concept and they model an intestinal crypt rather than a colonic crypt. Byrne and co-workers implemented a multiscale version of the Meineke model and applied it to a colonic crypt [36], [37]. By including an intracellular WNT-dependant cell cycle model they transformed the Meineke model to a niche-based model. They showed that the Meineke ‘pedigree’ model with immortal stem cells is inconsistent with the observation of niche succession (unlike their model), thereby essentially comparing a pedigree model with a niche model. However their focus was mainly on the effect of including multi-scale aspects in the Meineke model rather than comparing parameter estimates for the pedigree and niche models. Very recent work by Fletcher et al. [38] found that the stem cell niche hypothesis (related to the niche concept in our work) results in more realistic small intestinal crypt behavior than the immortal stem cell hypothesis (related to the pedigree concept of our work). Although Fletcher et al. also evaluate two hypotheses of crypt stem cell behavior, their main focus is on monoclonal conversion of the crypt, and most of their model analysis are done using only the stem cell niche hypothesis.

### Parameters

Avoiding the inclusion of too many free parameters (those whose values have to be estimated) is always a challenge in computational modelling. Fortunately many of the most important parameters in our model can be fixed from experimental findings (Tables 1 and 2). Specifically the cell cycle parameters (Table 1) and the crypt size parameters (Table 2) are based on the experimental results of the descending colon reported in [6] and [7]. However the cell cycle parameters reported in these studies only apply to a single group of proliferative cells, whilst our pedigree model assumes two groups of proliferative cells – stem cells and TA cells. Since convincing markers for stem cells have only recently been reported [13], [14] we assume that the cell cycle parameters reported in [6] and [7] predominantly apply to TA cells. The pedigree model is further informed by a system of Ordinary Differential Equations (ODE's). The steady state solutions of the ODE's together with the experimental data from [7] are used to estimate the stem cell division rate analytically (see Text S1).

**Table 1:** Cell cycle parameters.

Cell cycle phase	Stem cell value^*^	TA-cell value
G1	22.5	7.0
G2	6.0	1.8
M	1.5	0.5
S	20.0	6.2

Values used for the Smith-Martin cell cycle model (all values are given in units of hours). 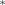
Values for stem cells are estimated using the ODE model.

**Table 2:** Fixed parameters.

Parameter	Unit	Value	Reference
Number of TA-cell generations (pedigree only)	Generations	5	[15] and best fit
Stem cells attached to the crypt base (pedigree only)	Boolean	TRUE	Model prediction
Number of circumference cells	Cells	22	[6], [7]
Number of length cells	Cells	32	[6], [7]
Mean cell radius	Microns	5	[69]
Spring constant over η	1/s	1×10^−7^	Estimated

Parameters fixed for both the pedigree and niche models. 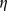 is a constant based on the area of contact and separation between cell i and the basement membrane (see Equation 5).

The other free parameters for both the pedigree (Table 3) and niche (Table 4) models relate to general remaining uncertainties in intestinal crypt biology. It is expected that these parameters will have a biological significance (Table 5). The effect of parameter variation on crypt behaviour is explored in a parametric study using our agent-based model. In order to identify the most desirable parameter values, we designed suitable measures of crypt performance (based on matching of experimental data as well as a computed statistic) to evaluate how well a set of parameters can match our performance criteria. We then explored the parameters in a parallel computing environment for both the pedigree and niche models. Some of the parameter estimates of our pedigree and niche models overlap. By comparing the patterns of optimal values found for these parameters we obtain interesting insights into similarities and differences of the two models. For example both models indicate that cell division is mostly in a vertical direction but only the niche model support lateral migration of cells. The niche model also resulted in a much more orderly separation between proliferative and mature cells as implied by Ki67 immunostain results. We extended our comparison by exploring how well each model responds to a variable demand in cell production. We assumed that a cell's most likely response would be to adapt its cell cycle time, hence this exploration was done by evaluating the sensitivity of each model to changes in cell cycle parameters.

**Table 3:** Pedigree free parameters.

Parameter	Unit	Values
Attractive force over *η*	m/s	0,0.5×10^−8^,1.0×10^−8^
Attractive force distance	Microns	0, 0.5, 1
Active migration	Boolean	TRUE, FALSE
Lateral migration	Boolean	TRUE, FALSE
Vertical division only	Boolean	TRUE, FALSE

Parameters explored for the pedigree model. 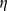 is a constant based on the area of contact and separation between cell i and the basement membrane (see Equation 5).

**Table 4:** Niche free parameters.

Parameter	Unit	Values
Only new cells mature	Boolean	TRUE, FALSE
Proliferation region	Fraction of crypt length	0.2, 0.3, 0.4, 0.5
Attractive force over *η*	m/s	0,0.5×10^−8^,1.0×10^−8^
Attractive force distance	Microns	0, 0.5, 1
Active migration	Boolean	TRUE, FALSE
Lateral migration	Boolean	TRUE, FALSE
Vertical division only	Boolean	TRUE, FALSE

Parameters explored for the niche model. Meaning of ‘Only new cells mature’ if true then only cells born in the non-proliferative region will be regarded as mature; if false then any cell that is in G1 and move out of the proliferative zone will mature. 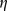 is a constant based on the area of contact and separation between cell i and the basement membrane (see Equation 5).

**Table 5:** Biological significance of free parameters.

Parameter	Biological significance	Reference
Attractive force	Cell-cell adhesion	[70], [71]
Attractive force distance	Cell filopodia extruding to each other so that cells not in physical contact can be attracted to each other	[70]
Active migration	Active migration or not?	[43], [72]
Lateral migration	Could cells migrate laterally or only vertically?	[42], [43]
Vertical division only	Some observation of crypt cells dividing vertically only has been reported	[44]
Only new cells mature	If false cells in G1 can be arrested into dormancy ( = mature)	This manuscript
Proliferation region	Extracellular regulation of cell fate, e.g. WNT gradient.	[73], [74]

After choosing parameter sets that gave a good fit to the performance expectation for the crypt, and following an evaluation of cell cycle sensitivity, the question arises as to how well each model could describe the observed LI data of [6] and [7]. Because our models were calibrated using performance criteria that excluded the traditional approach of fitting LI profiles, we were motivated to evaluate how well our calibrated model could match the experimental LI data. Interestingly the pedigree model match the LI data more satisfactorily. However, by adapting properties of each other, we show how a modified pedigree and niche model can both obtain good fits to the entire LI profile and simultaneously produce ordered crypts.

## Results

The stochastic phase in our modified Smith-Martin cell cycle model (see the Methods section) results in not only a range of cell cycle times but also in a variation of cell sizes between individual simulations. To account for this stochastic variation all our results are based on the mean of at least 30 simulation runs. From empirical studies we have found that 30 simulations sufficiently reduces variability without compromising the practicality of doing a parametric study.

### Model evaluation

The effectiveness of a particular model in simulating a healthy crypt can be assessed using a number of measures. The most important measure in this study is the total number of cells maintained in the crypt during steady state. Overabundant production of cells could potentially lead to crypt buckling [39], [40] and the consequential formation of adenomas. If too few cells are produced, the epithelial lining might tear, allowing bacteria and other harmful substances to enter the body. Different regions of the murine colon have been reported to have different sized crypts [7]. In this study we will focus on crypts in the descending colon that have approximately 700 cells [6], [7]. This number is calculated by multiplying the average number of cells along the length of the crypt (31.1) by the average number of columns per crypt (22.5). We will refrain from using half cell sizes and hence our virtual crypts are designed to be 32 cells in the length and 22 cells on the perimeter giving a total of 704 cells. Our crypt sizes are finely tuned (taking into account average cell size and overlap between cells) to allow for exactly 704 cells if the cells are perfectly arranged. We thus set a criteria to accept parameter sets that result in steady state cell numbers in a small 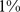 tolerance interval of 704. All the others are regarded as unsatisfactory steady state parameter combinations. This 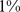 constraint comes immediately from considering the critical biological necessity for maintaining epithelial sheet continuity over the colonic mucosae. Unless the rate of production of crypt cells is tightly regulated, cells would accumulate on the colonic mucosae much faster than they are removed (indicated by large cell numbers in the case of our model with fixed crypt size), which is physiologically unsustainable. Alternatively if production is too low, the epithelial sheet would tear (indicated by fewer cells in our model), which is also physiologically unsustainable. If we were to model a portion of mucosal area that included multiple crypts, a less stringent limit on cell number and cell production rate would probably have been more appropriate since it is realistic to expect significant crypt-to-crypt heterogeneity in cell production rates.

The experimental results of [6] and [7] report two further measurements that can be used to evaluate model performance. These are the proportion of proliferative cells in the crypt and the crypt cell production rate (reported in units of cells/hour). The number of proliferative cells have been reported to be 200 so we will regard parameter sets that result in a 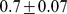 proportion of mature cells in the crypt as acceptable. We similarly allow for a 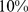 tolerance interval for the cell production rate giving the acceptance interval of 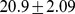 cells per hour. Note, these last two parameters have a larger tolerance level than cell numbers since the experiments used to determine them are more complex and hence more prone to uncertainty. We measure the cell production rate of our models by keeping track of the number of cells leaving the crypt every simulated hour. Since it is mostly mature cells leaving the crypt, we regard this measurement as the mature cell production rate. If this is not the case and too many immature proliferating cells occupy the functional region of the crypt, the crypt runs the risk of having too few functional cells capable of performing absorption or mucus secretion. Furthermore, increased biophysical tension from the growing proliferative cells in the upper crypt region could cause buckling. To facilitate the filtering out of crypts that produce abundant amounts of non-mature cells in the lumen we introduce the concept of mature cell order.

We define a mathematical expression to measure the order of each cell as:
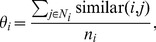
(1)where 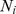 is the set off all neighbours of cell 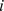 (the cells that are in contact with 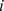), 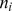 is the number of elements of this set, and



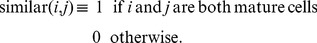
(2)The order of the mature cell population are simply the mean cell order of all 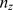 mature cells:



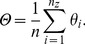
(3)


A perfectly ordered mature population will result in values near 1 (Note, an order value of 1 can never be reached in healthy crypts since there will always be contact between proliferating and mature cells). We will regard crypts with a mature cell order greater than 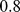 as acceptable, although we will give preference to crypts with higher order if needed, to rank cases that are similar based on our other 3 performance measurements. Preferring highly ordered mature cell populations is also motivated by the experimental observation that there is a clear separation between proliferating and non-proliferating cells [10], [11].

### Parameteric study results

**Figure 3:**
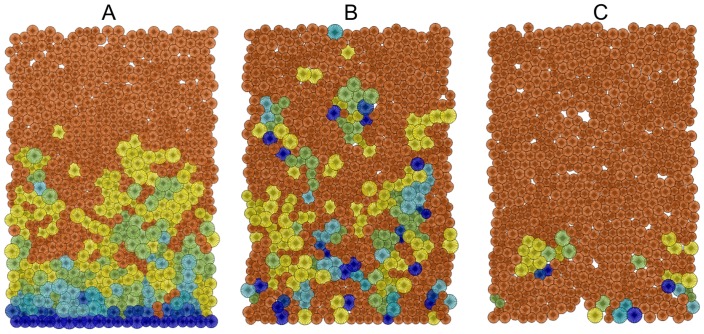
Stem cells (navy) are lost in the pedigree model if there is no attachment force to hold them in place. A: a snap-shot produced by the Repast modelling interface of a typical steady state cell distribution for the pedigree model crypt with stem cells attached to the base. B & C: when stem cell attachment is disabled stem cells are progressively transported out of the crypt and the crypt becomes a non-proliferating crypt full of mature cells. This state is likely to lead to crypt involution.

108 sets of parameters were explored for the pedigree model and 432 sets for the niche model. Our performance measures described above were calculated using time averaging for each simulation, as well as averaging over multiple simulations (at least 30). An immediate observation with the pedigree model is that an assumption requiring stem cells be attached to the base of the crypt is needed if they are required to remain in the crypt (Figure 3). If no horizontal migration is allowed, stem cells remain at the bottom of the crypt even without being attached, but if we allow lateral cell movement, and the stem cells are not attached to the base, then the stem cells are eventually transported out of the crypt and lost. Once crypts have lost all their stem cells, they consist only of non-proliferating cells (see Figures 3 B & C). The broader implication of this observation is that our multiple stem-cell pedigree model with stem cells physically attached to the base cannot explain niche succession [37]. However, niche succession does not refute the pedigree concept, since it could be explained by a pedigree model with more complex stem cell organisation. One such example would be a pedigree model with a single ‘reserve stem cell’ which replenishes a larger more active pool of stem cells as the need arise, similar to the dormant -and active hematopoietic stem cells observed in the bone marrow [2], [3], [41].

**Figure 4:**
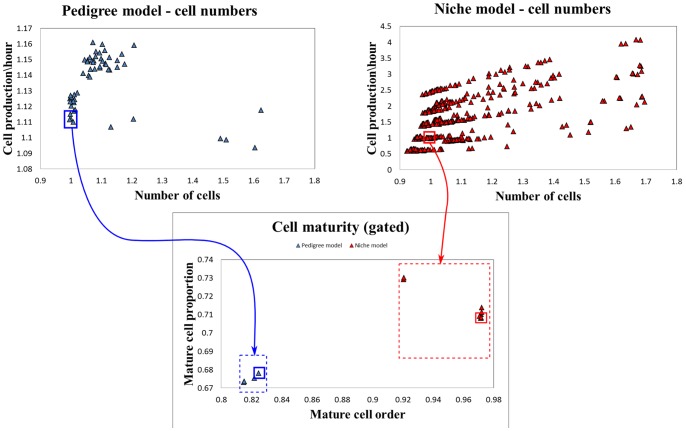
Choosing the best performing parameter sets. Scatter plots of normalised model performance for the pedigree (blue points) and niche (red points) models. Each point represents a simulation ‘run’ with a unique parameter set. We first ‘gate’ on the cell number and cell production performance criteria (left plots). These filtered runs are then plotted on a cell maturity performance scatter plot, from which the best performing runs can be identified. Gates are indicated in the figure by the blue and red rectangles, and are chosen such that preference is given to normalised performance values closer to 1.

The parametric study results can be summarised using scatter plots (Figure 4) to show the outcomes for each parameter set (called a ‘run’). For the pedigree model we chose the best performing parameter set by first gating on the *Number of cells* and *Cell production rate* (Figure 4A). As all the runs with normalised cell numbers less than 1.01 have a normalised *Cell production rate* outside our gating tolerance (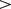
1.1), we had to raise the acceptable *Cell production rate* to 1.12. It turns out that the pedigree model has a propensity towards cell overproduction, an observation confirmed by our sensitivity analysis which shows insensitivity in cell production of the pedigree model to cell cycle time changes (see below). Five runs qualified for our gating criteria, and the one parameter that all these runs had in common was having no lateral cell migration (cells move in vertical bands – see Figure S1 A and Table S1). For further analysis we have chosen run 13 as representative of the selected group of five (Figure 4A right), based on the fact that run 13 had the normalised *Cell production rate* closest to 1, and it also had the smallest variance (not shown) in performance measurements amongst the group. The free parameter values (see Table 3) of run 13 are: Attractive force over 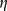: 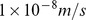; Attractive force distance: 0 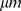; Active migration: FALSE; Lateral migration: FALSE; Vertical division only: TRUE.

For the niche model, gating on *Number of cells* and *Cell production rate* resulted in seven runs (Figure 4B). From these a final set of four were chosen based on a *Mature cell proportion* 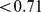. In general the niche model fitted our performance criteria better than the pedigree model and also resulted in more orderly crypts. Interestingly the best runs of the niche model support lateral migration (Table S2) – in contrast to the best runs of the pedigree model. There seems to be agreement that although migration in the crypt are mostly in vertical lines [42], lateral migration also occurs [43]. There also seems to be a trade-off in model performance between attractive (Attractive force and Attractive force distance) and repulsive (Active migration and Vertical division only) cell-cell contact parameters observable in Table S2. Larger combinations of attractive parameters, which result in slower migration of cells toward the top of the crypt, are always balanced out by activating one or both repulsive parameters. The influence of the two niche model specific parameters (Proliferation region and Only new cells mature) can be clearly observed in Figures S1 B & C. Perhaps not surprisingly, crypts with larger proliferation regions have a higher cell production rate but also have less order amongst mature cells. A similar pattern can be observed for crypts where Only new cells mature  =  TRUE, that is, only cells born in the non-proliferative region will be regarded as mature, cells that migrate into the non-proliferative zone will continue their proliferative cycle.

**Figure 5:**
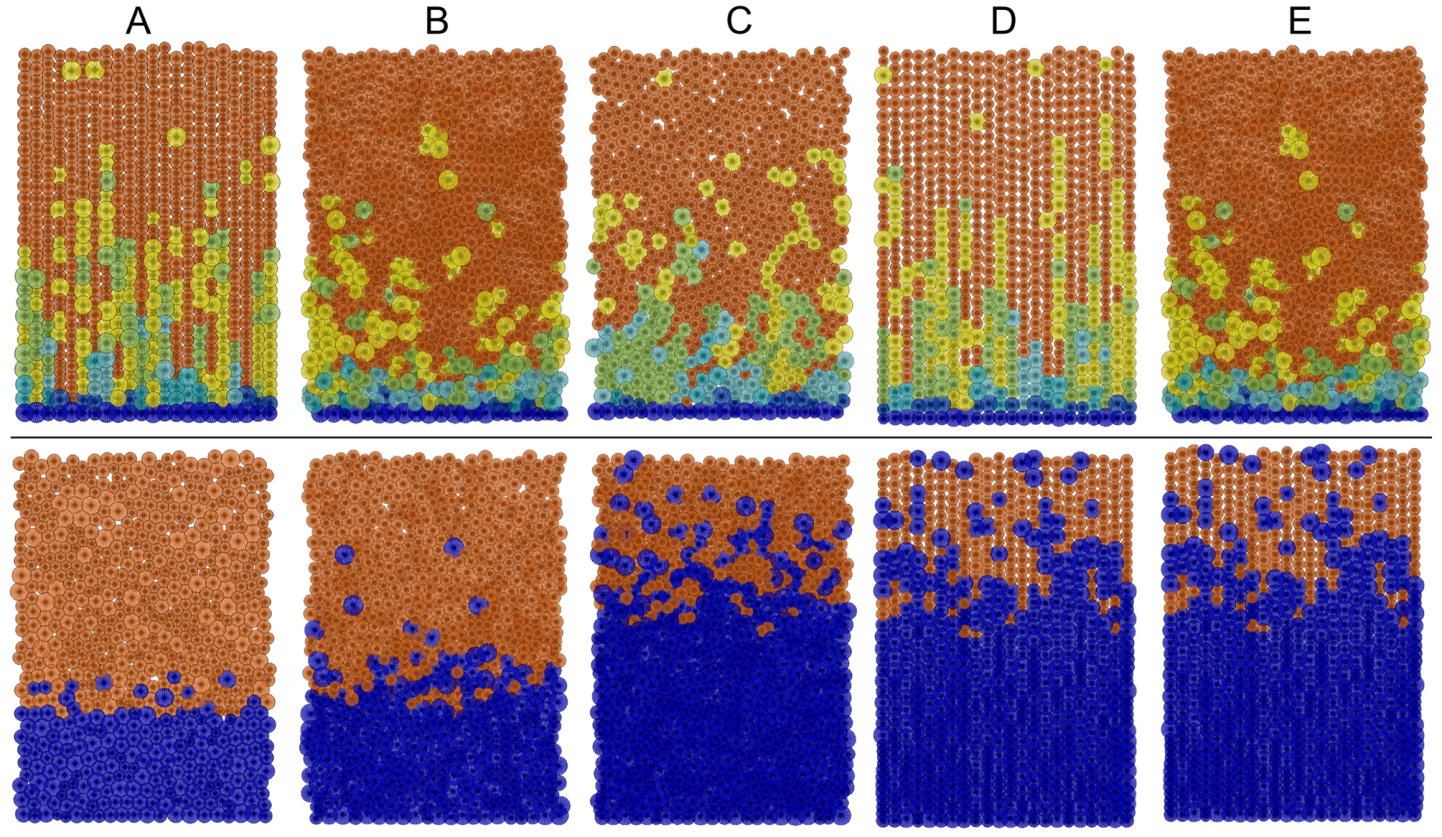
Visual snapshots of best and worst virtual crypts at steady state. A: Chosen best models. B: Worst performance on *Number of cells*. C: Worst performance on *Cell production rate*. D: Worst performance on *Mature cell order*. E: Worst performance on *Mature cell proportion*. Pedigree model results are shown in the top row and niche model results in the bottom row.

From the set of four best niche runs we chose run 296 as representative to use in our further analysis: Attractive force over 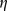: 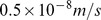; Attractive force distance: 0.5 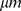; Active migration: FALSE; Lateral migration: TRUE; Vertical division only: TRUE; Only new cells mature: FALSE; Proliferation region: 0.3. For comparison we show visual snapshots of best and worst virtual crypts (both pedigree and niche models) at steady state in Figure 5.

**Figure 6:**
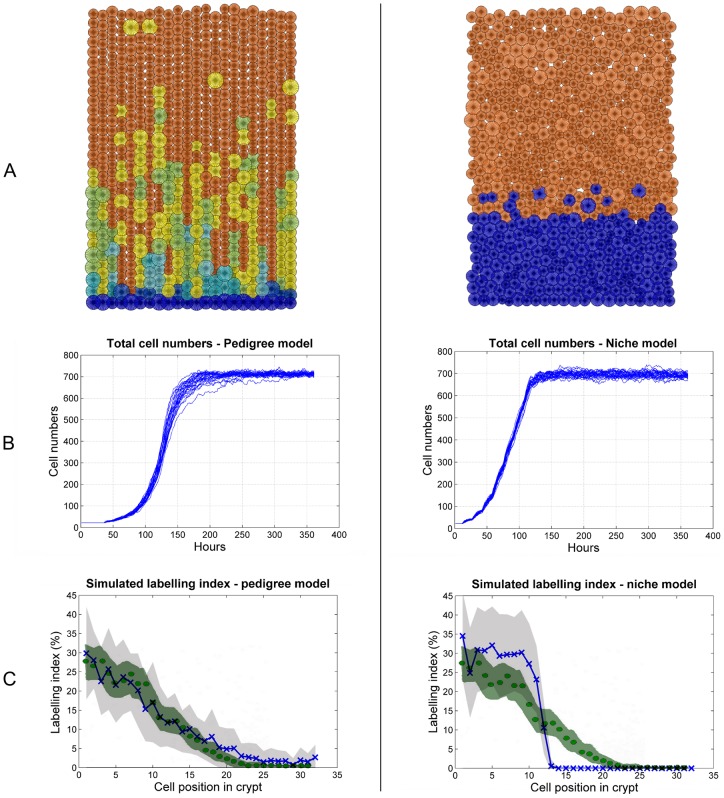
Simulation output of our chosen best performing pedigree (left panel) and niche (right panel) model. A: Visual snapshot at steady state. The fact that the pedigree model has no lateral cell movement is evident, whilst the extremely high ordering in mature cells and separation between proliferative and mature cells of the niche model are also clearly visible. B: Cell number output of each of the individual thirty simulations. It is evident how the pedigree model takes longer to reach a steady state. C: LI simulations (mean trajectory: blue line and X's; 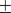 standard deviation: light grey shade) overlaid with experimental data from [7] (mean trajectory: green dots; 95% confidence interval: green shade.

A steady state snapshot of simulations using the chosen parameter sets together with the individual cell number trajectories are shown in Figures 6A & B. Clearly visible in Figure 6A is the lack of lateral migration and a tendency to disorder in the pedigree model (left plot). The high order within and sharp boundary between proliferative and mature cells observed in the niche model (right plot) is in agreement with what has been observed with Ki67 immunostaining experiments [10], [11]. The observed variation in mature cell sizes (orange cells) of the niche model are simply due to the stochastic variation in cell cycle times and do not relate to experimentally observed variation in the sizes of columnar and goblet cells [44].

Using the parameters of our chosen best models, we simulated clonal trajectories in real time (meaning the simulation output can be watched like a movie while the simulation is running, i.e. real time for the observer). These simulations are performed by marking 4 clones at the base of the crypt and then letting all descendants of the marked clones inherit the mark. Marked cells are then visualised as they migrate up the crypt. The clonal trajectory simulation results (Figure S2) show that models with lateral movements of cells are also successful in modelling the straight ribbon-like trajectories observed in the lab [45]. Gaps do appear in the ribbons of the lateral-enabled pedigree model however, providing further support for no lateral migration in the pedigree model.

### Sensitivity to cell cycle parameters

We have described our results for our virtual crypts at steady state. However during recovery from injury, the dynamic adjustment of the supply of mature cells in response to demand (fluctuations around steady state) becomes an important consideration. From a process engineering viewpoint we can also regard the crypt as a control system that has to respond efficiently to demands of more or less mature functional cells. For such a control system the question then arises: what variables are the best candidates for controlling cell production?

We propose the cell cycle duration parameters as a logical choice for controlling cell numbers and production in our models. By evaluating how sensitive our pedigree and niche models are to changes in cell cycle times (a fixed parameter so far) we could compare how well each model would respond to a fluctuating demand for mature cells. A model that is more sensitive to changes in the cell cycle times would exhibit better control through varying its cell cycle times. Analytical sensitivity measures like those used for differential equations cannot be applied in this case, because our two virtual crypt models are discrete stochastic models. Hence sensitivity analysis is estimated empirically. For our analysis we calculated the ‘Relative Sensitivity Function’ (see Equation 9 in the Methods section) of the four performance measures to changes in the G1 phase of the cell cycle (G1 refers to the Gap 1 phase of the cell cycle in which the cell increases its size up to a checkpoint where everything is ready for DNA synthesis). Thus we systematically varied G1 whilst keeping all other parameters constant and set to the chosen optimal values given above (run 13 for the pedigree and run 296 for the niche model). The results for single normal operating points (the cell cycle parameters given in Table 1) are summarised in Table 6.

**Table 6:** Result of sensitivity analysis.

Model	Adjusted parameter	Normal operating point (hours)	Cell number	Cell production	Mature cell proportion	Mature cell order
**Pedigree**	SC G1	22.5	−0.021	−0.546	0.227	0.085
	TA G1	7	−0.18	−0.166	−0.289	−0.146
**Niche**	G1	7	−0.452	−1.222	0.042	0.016

Normalised sensitivities for our four measures of performance, calculated using Equation 9.

From Table 6 we see that *Cell number* and *Cell production* are more sensitive to G1 (and hence can be controlled more effectively by changes in the cell cycle times) for the niche model than for the pedigree model. The *Mature cell proportion* has highest sensitivity to G1 in the pedigree model. There is however an opposite effect on the *Mature cell proportion* when adjusting stem cell cycle times compared to adjusting TA cell cycle times. Specifically, slower stem cell cycle times increase the *Mature cell proportion* (sensitivity +0.227), however slower TA cell cycle times decrease the *Mature cell proportion* at a similar rate (sensitivity −0.289). Hence, equal change in both the stem cell and TA cell cycle times will probably have little effect on the *Mature cell proportion*, as is the case for the niche model.

### Labelling index simulations

Having an explicit synthesis-phase in our cell cycle model enabled us to simulate LI experiments. A significant number of experimental LI results can be found in literature, although most are for the small intestinal crypt [8], [21], [46]–[48]. There seems to be some discrepancies between reported experimental results which create the impression that LI experiments are sensitive to the tissue and location within the tissue where cells are labelled. Fortunately the experimental results on which our parameter estimation were based report LI results for the descending colon [6], [7]. We thus had experimental LI data on the same region of the colon that we designed our models to represent.

Our LI simulations were performed as follows. At some point during steady state all cells in phase S of their cell cycle are marked (S refers to the synthesis phase of the cell cycle in which DNA replication occurs). This marker is transferred to descendants of the labelled cells and one simulated hour after marking was initiated the proportion of labelled cells at each crypt position (the centre of each cell determines its vertical position) is calculated. The results (average and standard deviation) of 30 stochastic numerical LI simulations with overlaid experimental data are shown in Figure 6C. Interestingly the pedigree model results match the data in the lower half of the crypt better, whilst the niche model is more effective in fitting the upper third of the crypt where the LI values are zero (and variation is less as well). A summary comparing these and other results for the pedigree and niche models is given in Table 7.

**Table 7:** Results summary.

	Pedigree model	Niche model
**Parametric study results**		
Attractive force	1.0×10^−8^	0.5×10^−8^m/s
Attractive force distance	0 μm	0.5 μm
Active migration	FALSE	FALSE
Lateral migration	FALSE	TRUE
Vertical division only	TRUE	TRUE
Proliferation region		0.3
Only new cells mature		FALSE
**Cell cycle sensitivity**	Less sensitive	More sensitive
**Ki67 data fit**	Poor fit	Good fit
**LI data fit**	Good fit	Poor fit
**Clonal trajectories**	Gaps appear if lateral migration allowed	No gaps even if lateral migration allowed

**Figure 7:**
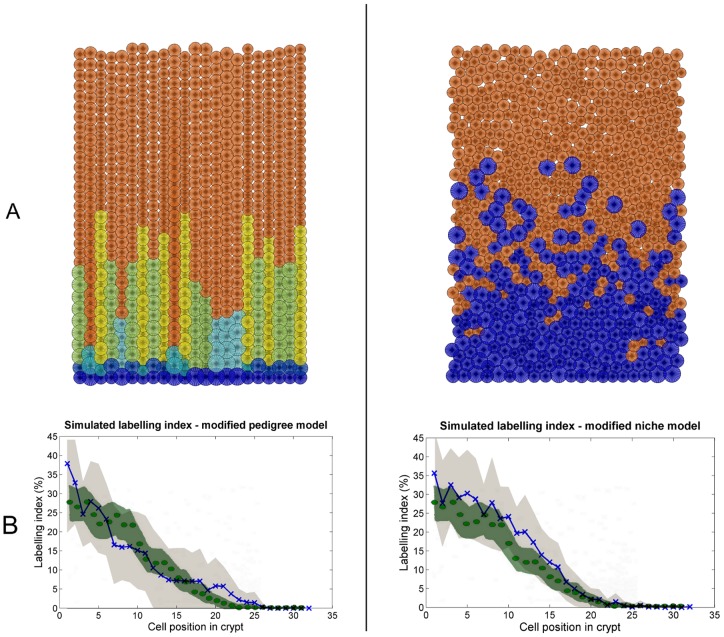
Simulation output of the adapted pedigree (left panel) and niche (right panel) model. A: Visual snapshot at steady state. B: LI simulations (mean trajectory: blue line and X's; 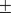 standard deviation: light grey shade) overlaid with experimental data from [7] (mean trajectory: green dots; 95% confidence interval: green shade.)

From our simulation results in Figure 6C (right panel) we can see that the abrupt decrease in the LI profile of the niche model is not smoothed out by averaging over multiple crypts, but it is most likely due to the clear separation of the proliferative and non-proliferative zone (Figure 6A right plot). This separation has been strongly supported by the Ki67 immunostaining results, in contrast to the large degree of intermingling between proliferative and mature cells observed in the pedigree model (Figure 6A left plot) which is not in agreement with the Ki67 results (but fits the LI profile very well). These observations raise the issue about whether the earlier LI results [6], [7] are consistent with the more recent Ki67 immunostains [10], [11]. As can be observed in Figure 7, both the pedigree and niche model can indeed be adapted to fit the LI profiles and Ki67 results simultaneously. To achieve this, for the pedigree model all stochastic variation in the cell cycle times were removed so that cells cycle at a deterministic unsynchronised (out of phase) rate. For the niche model more spatial stochastic variation was introduced by adopting a position-dependant probability distribution function that controls whether a cell matures or not. In this case, LI and Ki67 data are now reasonably consistent with each other.

## Discussion

We have developed agent-based models of ‘virtual colonic crypts’ for the mouse. Our models allow for single cells to be represented and displayed in a simulation, and hence also enable visual observation of the effects on the whole crypt due to changes on a cellular level. Like any model of a real system, there are simplifying assumptions (e.g. a 3D crypt is mapped to a 2D geometrical domain) and the parameters within the model actually represent the macroscopic effects of many complex processes occurring at smaller length scales (e.g. viscous dissipation by multiple cell-cell and cell-basement membrane interactions).

For any quantitative crypt model based on mechanistic principles, one can hypothesize two extreme states: (i) all parameters in the model are ‘predetermined’ and so fixed in space and time, and (ii) all parameters in the model are variable over space and time. Between these extremes there is a continuum containing an infinite number of models with varying degrees of fixed or variable parameters. In reality, it is most likely that an actual colonic crypt maps onto this continuum of models somewhere between these two extremes. That is, some parameters in an optimal quantitative model will be relatively fixed (i.e. only a relatively small number of cell phenotypes are accessible from the stem cells located towards the base of the crypt) while other parameters in an optimal model will be highly variable (i.e. dependent upon environmental conditions). For the models of the mouse crypt that we have developed, we can map onto this continuum the features of what biologists describe as the ‘pedigree’ and the ‘niche’ model (we note in passing that there is no fixed definition of these terms in the biological literature). Thus our definition of a ‘pedigree model’ has immortal cells identified as stem cells and a fixed number (five) of cell divisions by a TA cell before it differentiates into a mature cell. On the other hand our ‘niche model’ identifies whatever cell (s) are at the bottom of the crypt as stem cells, and has a variable number of cell divisions before cells mature, depending on environmental cues (see Figure 2).

The morphology and dynamics of murine (rodent) colonic crypts have been studied in considerable detail over many years [44], [49]–[52]. Morphology and kinetic data are available for human colonic crypts [52], [53] and there are 2–4 times as many cells in human colonic crypts as the equivalent crypts in mice. Whilst the total cell numbers and individual cell volumes are larger in the human crypt, the crypt morphology, cell types, and the positions of proliferating cells are similar in the two systems [54]. The number of stem cells associated with murine colonic crypts has yet to be been determined. Modern measurements on the number of stem cells at the base of murine small intestinal crypts [55] and the orientation of the cellular events associated with movement of cells at the base of the crypts [56], suggest that it is timely to attempt similar measurements in human crypts. It should be noted that the size of crypts (i.e. height and cellularity) are different at different positions in the colon (proximal, mid and distal), the reasons for these differences may become apparent as the simulations of the type we report progress to include an analysis of the parameters which influence crypt height and corresponding cellularity. We have conceived the colonic crypt as a ‘cell factory’ that needs to produce mature cells to ensure a continuous sheet of cells covers the mucosal surface served by a crypt. Based on experimental observations, we have set down desirable ‘output specifications’ for our cell factory, when operating under typical conditions for a healthy laboratory mouse. The output specifications are: number of cells in the crypt, cell production rate, proportion of mature cells and a mature cell-order measure. The first two specifications are quantitative measures that ensures that sufficient cells are produced to enable coverage of the mucosal surface. The third and fourth specifications are measures of cell quality exiting the mouth of the crypt. The cell quality measures presumably ensure that the cells are capable of performing their intended functions while on the mucosal surface.

We first asked the question: For a target crypt size and geometry, for our cell cycle model and for our rules defining pedigree and niche models, are our pedigree and niche models capable of meeting the desired output specifications? To test this question, we began by ranking the importance of the crypt output specifications, placing highest importance on cell number and an adequate production of cells per hour, then gave next highest weighting to cell maturity and finally assigned the lowest weighting to cell ordering. We implemented a search, within a parameter space, for optimal models that met these specifications using a statistical ‘gating process’ based on our rankings (Figure 4). Our gating strategy thus proceeded by first choosing the parameters that result in the best performance in terms of cell numbers and production. From these we then chose the parameter values based on our two cell maturity performance measures. This process is one of many possible ways for identifying near optimal solutions from the hundreds of possible models – the gating method is simple and convenient for our purposes here.

We found that both the pedigree and niche models can meet the output specifications described previously. But there are some interesting differences between the two models and the different strengths and weaknesses of each model became clear. The niche model results in much more ordered organisation of mature cells and is effective in achieving a clear separation between proliferative and mature cell compartments along the crypt (Figure 6A right plot), corresponding to what has been observed in the Ki-67 immunostains [10], [11]. Other niche based models exhibit similar output [36], [37]. The pedigree model resulted in much less orderly crypts and also in general do not match the cell production criteria as well as the niche model. The niche model also tended to reach steady state at a faster rate (Figure 6B), meaning it is likely to restore pertubation from the steady state more effectively than the pedigree model.

During our gating process cell cycle parameters were fixed as given in Table 1. It is only during sensitivity analysis (after the normal operating points were determined using our gating process) that cell cycle parameters were varied. Analysing the sensitivity of the pedigree and niche model to changes in cell cycle times provided us with a method to evaluate how well each model would perform under adverse conditions. This method is based on the premise that when demand for mature mucosal cells change during toxic shock or injury, proliferating cells are likely to respond by adjusting their cell cycle rates. The results (Table 6) show that the niche model (which has higher sensitivity) would more rapidly respond to changes in cell production demand than the pedigree model.

We further evaluated our ‘optimal’ pedigree and niche models on data not used during the optimisation process, namely LI experiments. These LI simulations indicate that the pedigree model produces more biological realistic results in the bottom part of the crypt, whereas the niche model compared more favourable with data in the upper part of the crypt (where there is also less noise in the experimental data). However, we have also shown that with small adjustments a pedigree model can produce an ordered crypt, and a niche model can fit the LI profile (Figure 7). To obtain these outcomes, for the pedigree model we assumed zero variability in cell cycle times, and for the niche model we assumed a position-based probability distribution function (PDF) for a cell's decision to stop proliferation. Of interest here is that these adjustments move each model towards each other on the aforementioned continuum of models. The deterministic cell cycle modification to the pedigree model effectively adds external controls (or reduces internal decision making) – a niche model property. Similar, the PDF-based maturity rule added to the niche model gives some ‘decision making powers’ to the cell – a pedigree model property.

## Conclusion

Computational modelling can enhance the understanding of the mechanisms by which cell organisation in the crypt is achieved, as we have shown. Our study provided theoretical insights into some of the still unresolved [26] fundamental questions of crypt cell organisation. How stem cells divide (symmetric or asymmetric) is closely tied to the pedigree (asymmetric) and niche (symmetric) paradigms. All indications are that stem cells can adjust their division regime (symmetric or asymmetric) according to demand [57] and that there exists more than one stem cell population with a hierarchical relationship in the intestinal epithelium [58], [59]. First there is a quiescent group of stem cells identified by the marker Bmi1 [14] which contributes little to daily homoeostasis but rapidly proliferate upon injury [59]. Second the marker Lgr5 [13] which identifies a mitotically active group of stem cells that derive from the ‘fundamental’ Bmi1+ stem cells [58], [59]. Interestingly a very similar hierachical relationship among stem cells have been identified in the bone marrow (dormant and active hematopoietic stem cells) [2], [3]. Essentially these findings implicate that cell organisation in the crypt is based on a combination of the pedigree and niche concepts. Taken together, our results support this idea.

However, much laboratory work remains to be done to further our knowledge of where exactly reality lies on the continuum of theoretical models. In this quest our theoretical results can guide new biological experiments. From our simulations under the immortal strand hypothesis (i.e. the pedigree model) we found that attachment of stem cells to the base of the crypt is a critical assumption, otherwise all stem cells are eventually lost (see Figure 3). This implies that an in depth experimental study to confirm whether stem cells do in fact remain attached to the basement membrane or not, might shed light on the immortal strand hypothesis. For example experimental results that show stem cells are not attached to the base of the crypt will provide strong evidence against the immortal strand hypothesis in colonic stem cells (this reasoning is referred to as ‘modus tollens’ in propositional logic).

We further propose that a comprehensive LI study in the gut could prove especially useful. Many examples of LI profiles can be found in literature but we expect that a multi-term (short and long) and multi-location LI study in the gut could provide more answers than the small-scale LI experiments that have been performed to date.

## Methods

Our computational framework is implemented in Java using the Repast agent simulation tool-kit [60] which provides an intuitive interface to interact with and distribute our models (see Figure S3). We have taken care to optimise our implementation of the crypt geometry, cell shape representation and mechanical model algorithms in the framework to such an extent that our simulations can be visualised and interacted with in real-time on a single workstation or laptop. These interactions include starting, pausing and stopping the simulations as well as changing model parameters and switching between different visualisation modes. Moreover, the ability to run models in batch mode without visualisation further improved the speed of simulations and rendered our computational framework useful as a tool for small parameter exploration studies.

### Crypt geometry representation

The most realistic implementation of a cell in an individual-based model would be a 3-dimensional representation of the cell shape as well as the crypt. The recent model of the small intestinal crypt [61] where cells are modelled as spheres, is an example of such an implementation. 3D models are however quite slow hence we decided on a 2D representation. Since the epithelial sheet of a crypt is a monolayer of cells, it is possible to model the geometry of the crypt in a 2D space without loss of individual cell granularity. We thus visually represent the crypt geometry as a rectangular sheet of interacting cells with periodic borders to the left and right. Cells are free to move to any continuous spatial position in this 2-dimensional space and hence the model can be classified as a lattice-free model [34]. One resultant artefact of this approach is that the implied 3D shape is cylindrical and hence there are potential inaccuracies in the crypt bottom representation. However there has been previous models which successfully modelled the crypt geometry on a 2D rectangular space [21], [62].

### Cell representation

**Figure 8:**
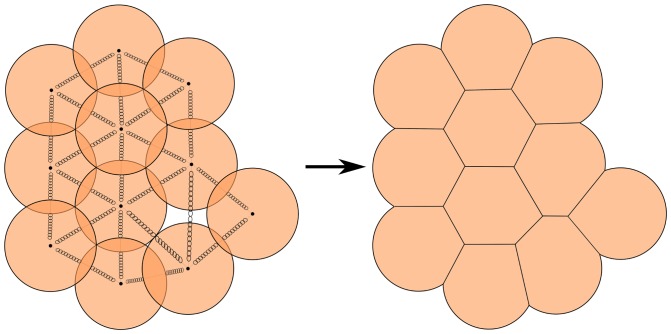
Cell shape representation. Representing cells as elastic 2D spheres via a linear spring model (Hooke's law). The implied cell shapes are shown on the right.

Voronoi tessellation has proven to be a realistic 2D mathematical representation of crypt epithelial cells [21], [36], [37]. Another cell shape representation is the vertex model [36]. Both the Voronoi and Vertex models tend to be computationally more expensive however than our 2D disc representation of individual cell shapes (Figure 8). Cells shape is maintained by internal springs and when cells overlap, they exert contact forces on each other. Cells which are not overlapping can also pull towards each other (filopodia) and our model includes a parameter (Attractive force distance) to explore this possibility as well. The magnitude of the force is calculated using Hooke's law (see below).

### Cell cycle

Our cell agents implemented a stochastic cell cycle model based on the Smith-Martin (S-M) model [63]. The S-M cell cycle model allows cells to be in one of two phases. At birth cells are in phase A and then transition to phase B with constant probability. The time spent in phase A is thus non-deterministic, whereas the time spent in phase B is deterministic and represents DNA synthesis, Gap 2, and mitosis of the cell cycle. The cell cycle parameters used in our simulations are summarised in Table 1. In addition to these four cell cycle phases we also have an (implicit) implementation of the G0 phase in the form of a boolean function which indicates whether a cell is cycling or not. Cells which are not cycling (the orange colored cells in Figures 3, 5, 6, 7) are in G0. The other cells can be in any one of the four phases given in Table 1. Since phase A consists entirely of G1 the probability of leaving phase A is determined by the average duration of G1 (7 hours for TA cells). In this case this probability would be 1/7 every hour, which results in an exponentially distributed duration of A with mean 7 hours.

### Cell growth

Our agent-based implementation of cells allows us to model cell growth explicitly. At the end of phase B the cell divides into two daughter cells of equal size (half the size of the mother cell at time of division). However the standard S-M cell cycle model do not take account of space and hence cell size and growth. We thus extended the S-M cell cycle model to a spatial version where cells grow during the G1 and G2 phases of the cell cycle. We assume that on average a cell would grow to double its volume (the target volume) before it divides, otherwise the average cell size would drift to zero or infinity. We further assumed that the stochastic phase of our S-M cell cycle model represents G1. The growth rate of cells is set to a constant rate such that the volume is increased in equal increments over G1 and G2. Hence all cells have exactly the same growth rate, except between stem cells and TA cells which have different cell cycle times. However not all cells will grow for the same time – hence we get different sized cells. Whilst this approach is sufficient for most cells on average, excessively large and small cells are observed on a regular basis. The main cause stems from the fact that a cell does not know when it will leave G1 so its impossible to adapt a faster or slower growth rate for shorter or longer G1 durations respectively. We thus define a minimum and maximum cell size. Cells below the minimum will not leave G1 and cells that have reached the maximum will stop growing even though they are still in G1 or G2. The average radius is assumed to be 5 microns, hence average radius when a cell divides (area doubled) is 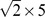. The maximum radius was chosen to be slightly larger: 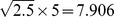. The minimum radius contributes to the condition for when the cell can leave state A/G1. The cell will then grow larger than this minimum during the short G2 phase. So the minimum radius was chosen to be 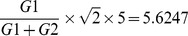.

### Model implementation

We adopt a process engineering view of the crypt as a ‘mature cell factory’. From this perspective the most important function of the healthy crypt is to produce enough mature (i.e. fully functional) cells to cover the portion of intestinal wall which it serves on the mucosal surface, with a supply rate of new cells that accommodates adequate cell turnover/renewal. Hence we do not explicitly model the crypt as a biological system as such, but instead model a mature cell factory that has clearly defined performance constraints. Our engineering approach enables the crypt to be given a well defined behaviour – but raises the issue of how mature cells should be defined. The definition of mature cells in this study are tightly linked to the pedigree and niche paradigms explained earlier. That is, in the case of the pedigree model mature cells are the descendants of the 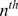 TA-cell generation. In the case of the niche model the environmental cues in which a cell finds itself will instruct the cell to be mature or not. In both cases mature cells do not divide or grow, but all other cells are proliferative.

Each simulation is initialised with only one row of cells at the base of the crypt. The ‘virtual crypt’ is grown from that one row until cell numbers reach a steady state. Each simulation differs in the time it takes to reach a steady state and hence we devised a method to detect when the simulation is at steady state. Our method is based on the rate of change in cell numbers (also called the cell number gradient) which we measure using a 12 hour moving average window. The initial gradient is zero whilst the first row of cell goes through the cell cycle. After a transient period the cell number gradient becomes positive as cell numbers increase and no cells are lost until the crypt has been fully grown. When the gradient returns to zero we assume that steady state has been reached, and the simulation is continued for at least 3 (simulation) days. All reported measurements are taken over this 3 day period.

In the pedigree model the bottom row of cells are assumed to be stem cells. They divide asymmetrically into another stem cell and a TA1 cell (see Figure 2A). The TA1 cells goes through a number of symmetric divisions before producing two mature cells. The stem cells and TA cells have different cycling rates (TA cells cycle faster) and we visualise the different cell types using the colour scheme depicted in Figure 2A.

For the niche model we define the proliferation region as a fraction of the length of the crypt, measured from the bottom of the cylindrical crypt representation. We distinguish only between proliferative cells and mature cells (see Figure 2B) and production of mature cells are modelled in one of two schemes. We either assume only proliferative cell that divides outside the proliferative region will produce two mature cells, or we assume that any cell that has not reached the S phase of the cell cycle and move out of the proliferative region will mature (see the Only new cells mature parameter in Table 4).

### Mechanics

At each time interval we assume that the forces executing on each cell are in equilibrium [64]:
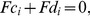
(4)with



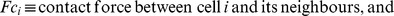


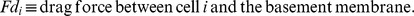



We approximate the drag force on cell 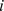 by
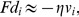
(5)where 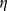 is a constant based on the area of contact and separation between cell i and the basement membrane (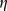 is assumed constant for all cells), and 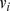 represent the relative velocity between cell 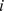 and the basement membrane.

The contact force between two cells with radius 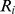 and 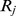 respectively, is based on a linear spring constant model (Hooke's law) and is calculated as follows
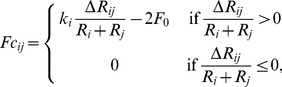
(6)where 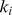 is the spring constant, 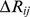 is the overlap between the cells, 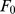 is an attractive force between overlapping cells, and 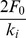 the equilibrium overlap (the 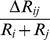 intercept). The size of the attractive 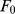 is used to represent changes in the strength of the cell-cell adhesion. We further assume that each cell has the same spring constant 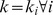, so if 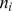 represent the number of cells in contact with cell 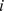 we have
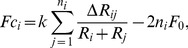
(7)

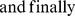




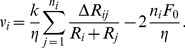
(8)


At each small time step, 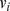 is calculated for all cells, after which the displacement of each cell is solved using the Verlet algorithm [65]. We investigated the performance of the Verlet algorithm in our models for various time step (simulation tick) sizes. Smaller time steps result in a more accurate approximation of cell displacement but also slows the simulation down. If the time steps are too large, the Verlet algorithm becomes unstable. We found that a time step of 10 seconds results in sufficiently fast simulations without compromising numerical stability of the Verlet algorithm.

### Relative sensitivity function

The relative sensitivity function is a dimensionless, normalised sensitivity measure of a function to changes in parameters at their normal operating points [66], [67]. The relative sensitivity of the function 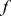 to the parameter 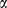 at the normal operating point (NOP) is given by
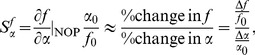
(9)where 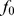 and 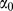 is the function and parameter at their NOP values respectively.

## Supporting Information

Archive S1 **The Repast Simphony 2.0 format crypt model files and documentation.**
(ZIP)

Figure S1 **Pedigree and niche model performance scatter plots.** Cell number measures are shown on the left and cell maturity measures are shown on the right. A: Pedigree model results, differentiating runs based on Lateral migration. Runs with Lateral migration = FALSE (red) performed better. B: Niche model results, differentiating runs based on Proliferation region. Proliferation regions of 0.2 and 0.3 tended to outperform the other clusters. C: Niche model results, differentiating runs based on Only new cells mature. From these two plots it is clear that Only new cells mature causes the distinctive bands on the niche performance plots.
(TIFF)

Figure S2 **Simulating clonal trajectories.** A: Pedigree model with no lateral migration. B: Pedigree model with lateral and vertical cell movement. C: Niche model with lateral and vertical cell movement.
(TIFF)

Figure S3 **Our interactive modelling interface.** A: Users have full control of model parameters, some of which can be changed during runtime (for example labelling cells in s-phase, or visualising the force gradient). B: A different view which shows the amount of force experienced by cells in the crypt.
(TIFF)

Table S1 **Pedigree best parameters.** The qualifying parameter sets (runs) for the Pedigree model when gating on cell numbers and cell production rate. A-force: Attractive force, AFD: Attractive force distance, A-Migration: Active migration, L-Migration: Lateral migration, V-division: Vertical division only.
(TIFF)

Table S2 **Niche best parameters.** The qualifying parameter sets (runs) for the Niche model when gating on cell numbers and cell production rate. P-region: Proliferation region, A-force: Attractive force, AFD: Attractive force distance, A-Migration: Active migration, L-Migration: Lateral migration, V-division: Vertical division only.
(TIFF)

Text S1 **The system of ODE**'**s that describe the pedigree model.**
(PDF)
