# Supplementary material for: Cell Organisation in the Colonic Crypt: A Theoretical Comparison of the Pedigree and Niche Concepts
Source: PLoS One. 2013 Sep 12;8(9):e73204. doi: 10.1371/journal.pone.0073204 (PMC3771985; doi:10.1371/journal.pone.0073204)
Supplement: Archive S1 — The Repast Simphony 2.0 format crypt model files and documentation. (ZIP) [file pone.0073204.s001.zip › RepastS installation instructions.pdf]

2013/07/02

Installing the model launcher Application

The crypt model can be opened with the Repast Simphony 2.0 interface which can be downloaded from http://repast.sourceforge.net/. However, we created a special version of the Repast Simphony suite that can be used easily to load and execute various models. This application launcher needs to be installed before models can be opened. To run Repast the Java Runtime Environment is required (JRE 1.6. Note JRE 1.7 not working at time of writing).

1. Download 'RepastS Launcher.zip' from https://docs.google.com/file/d/0B137b5scHLa4UHVWR2N4UUpaR2M/edit?usp=sharing
2. Extract 'RepastS Launcher.zip' and navigate to the 'Launcher' subdirectory. Double click on 'RepastS_setup_1.1.jar' (or type 'java –jar RepastS_setup_1.1.jar' on linux/mac systems).

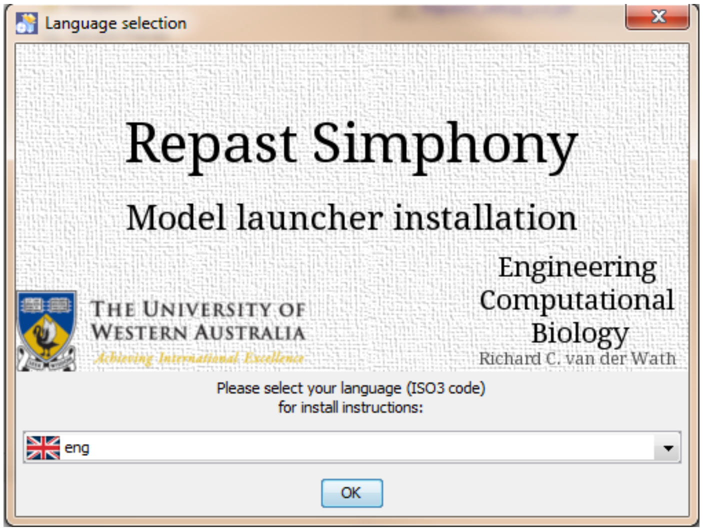

3. Follow the installation steps, click on ‘Done’ in the final step.

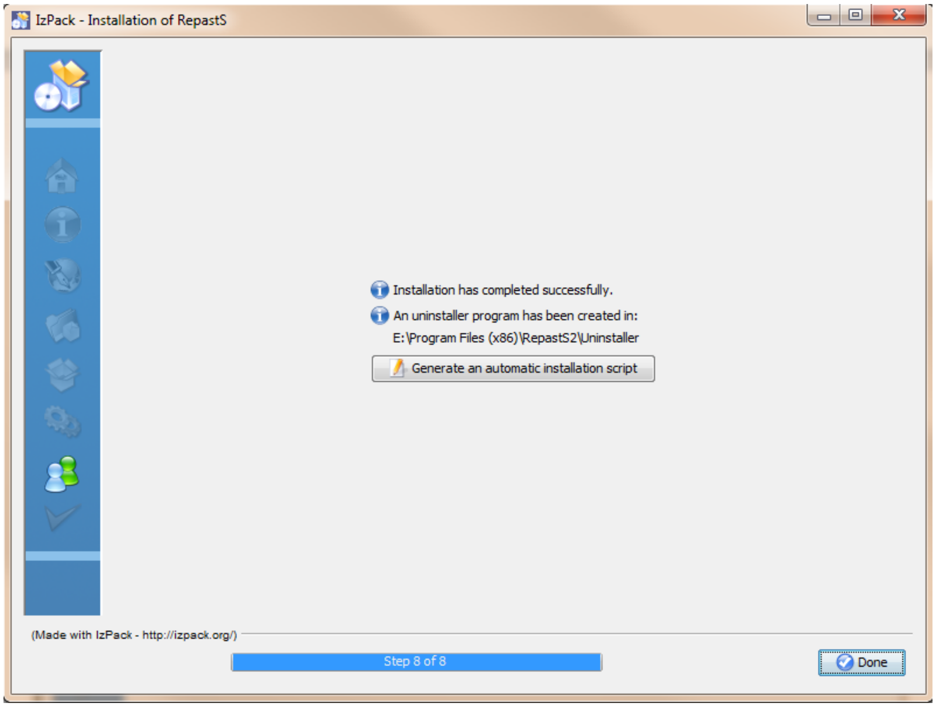

4. Mac OSX users only: download osx_libs.zip and extract the archive. Copy the 4 extracted .jnilib files to $REPAST_INSTALLFOLDER\RepastS\lib\.

To run a model

1. Start the RepastS launcher application. This can be done in a number of different manners:
   - From the start menu (if added during install)
   - Excecute $REPAST_INSTALLFOLDER\start_model.bat (windows) or $REPAST_INSTALLFOLDER\start_model.command (linux or mac)

2. You should see the following window

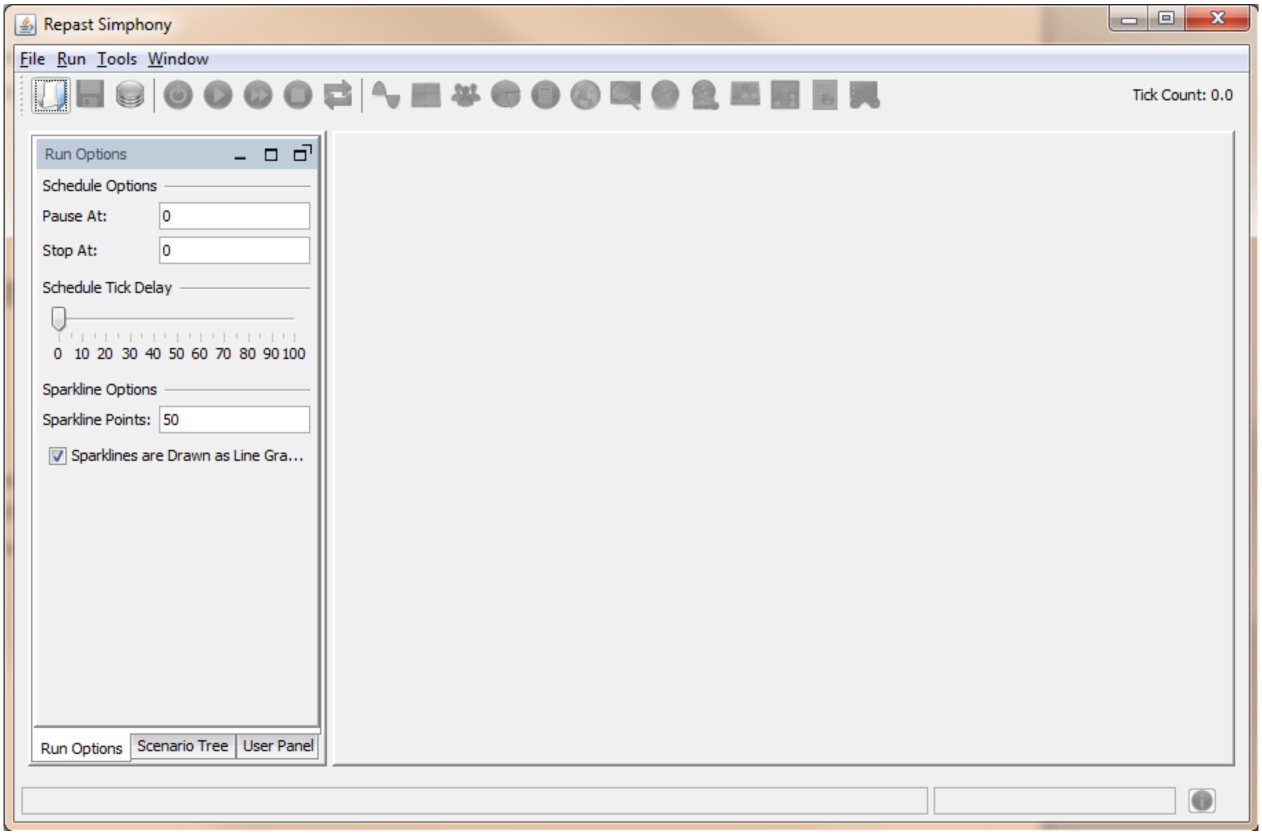

3. Click on the 'Open' icon in the top left corner, then navigate to the .rs folder of your Repast Simphony model

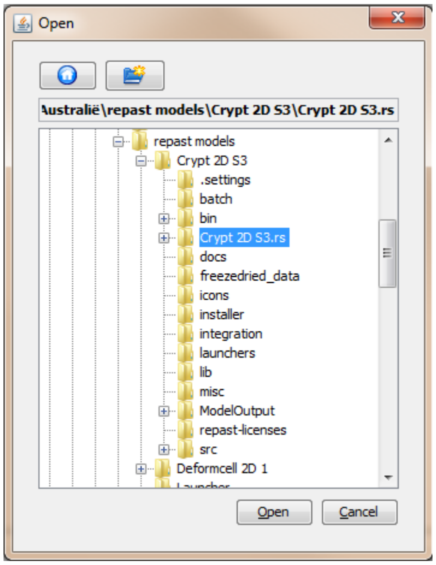

4. Once the model is loaded your screen should look something like

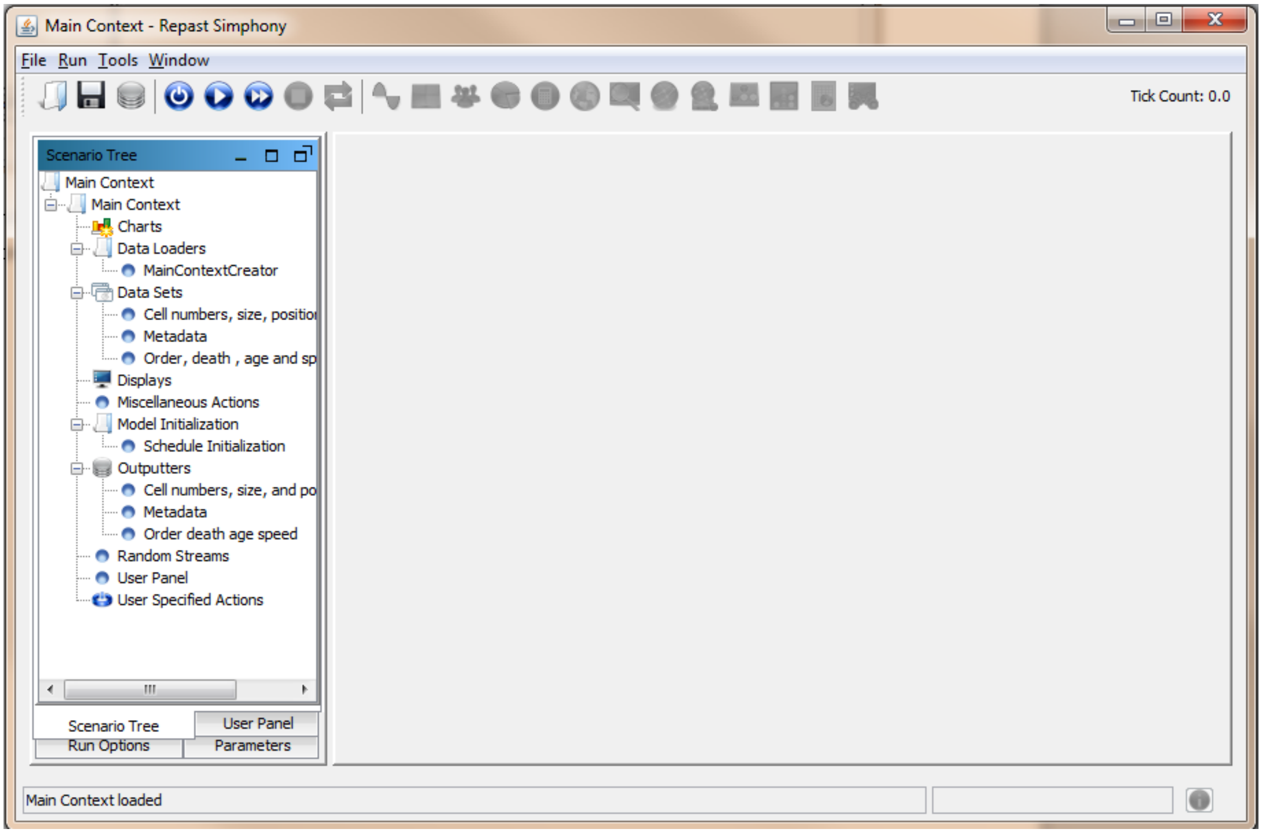

5. You can now init, run, pause, step, stop, reset the model using the blue buttons at the top or the options from the 'Run' menu.
6. To the left there are a number of panels where parameters and run options can be set. For full details, see the Repast S documentation.
