# Supplementary figures and images for: Cell Organisation in the Colonic Crypt: A Theoretical Comparison of the Pedigree and Niche Concepts
Source: PLoS One. 2013 Sep 12;8(9):e73204. doi: 10.1371/journal.pone.0073204 (PMC3771985; doi:10.1371/journal.pone.0073204)

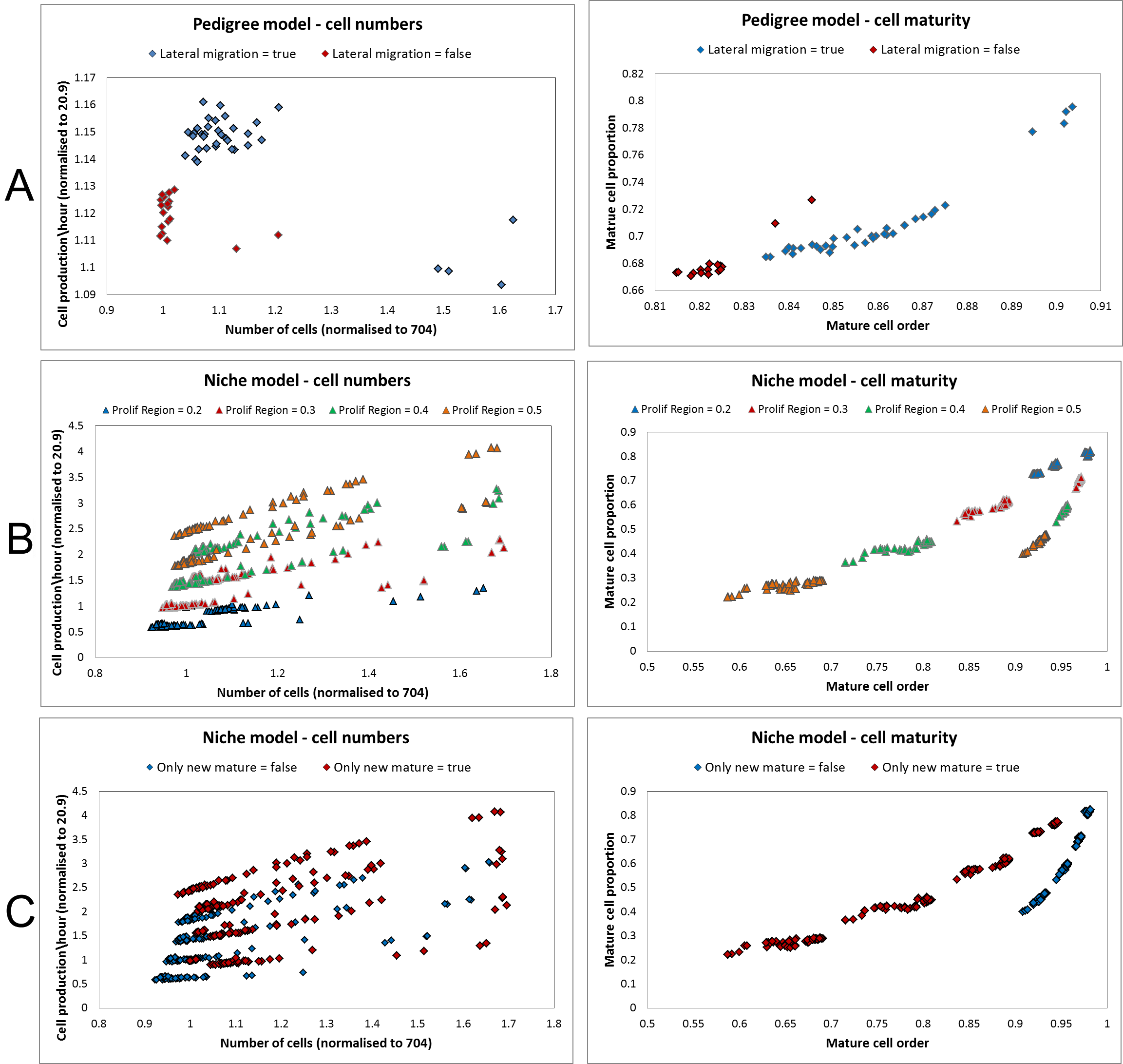

Supplement: Figure S1 — Pedigree and niche model performance scatter plots. Cell number measures are shown on the left and cell maturity measures are shown on the right. A: Pedigree model results, differentiating runs based on Lateral migration. Runs with Lateral migration = FALSE (red) performed better. B: Niche model results, differentiating runs based on Proliferation region. Proliferation regions of 0.2 and 0.3 tended to outperform the other clusters. C: Niche model results, differentiating runs based on Only new cells mature. From these two plots it is clear that Only new cells mature causes the distinctive bands on the niche performance plots. (TIFF) [file pone.0073204.s002.tiff]

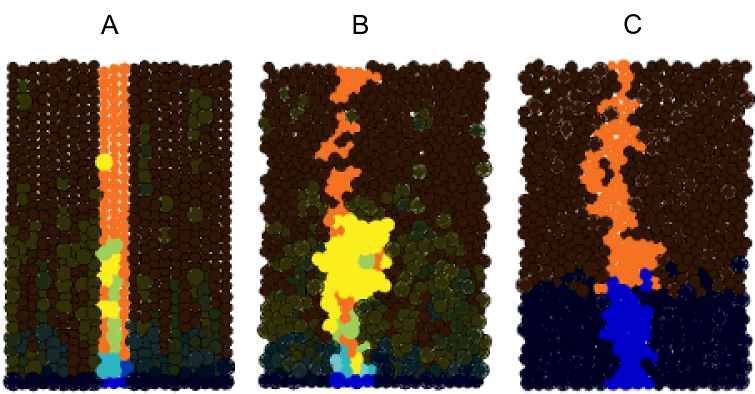

Supplement: Figure S2 — Simulating clonal trajectories. A: Pedigree model with no lateral migration. B: Pedigree model with lateral and vertical cell movement. C: Niche model with lateral and vertical cell movement. (TIFF) [file pone.0073204.s003.tiff]

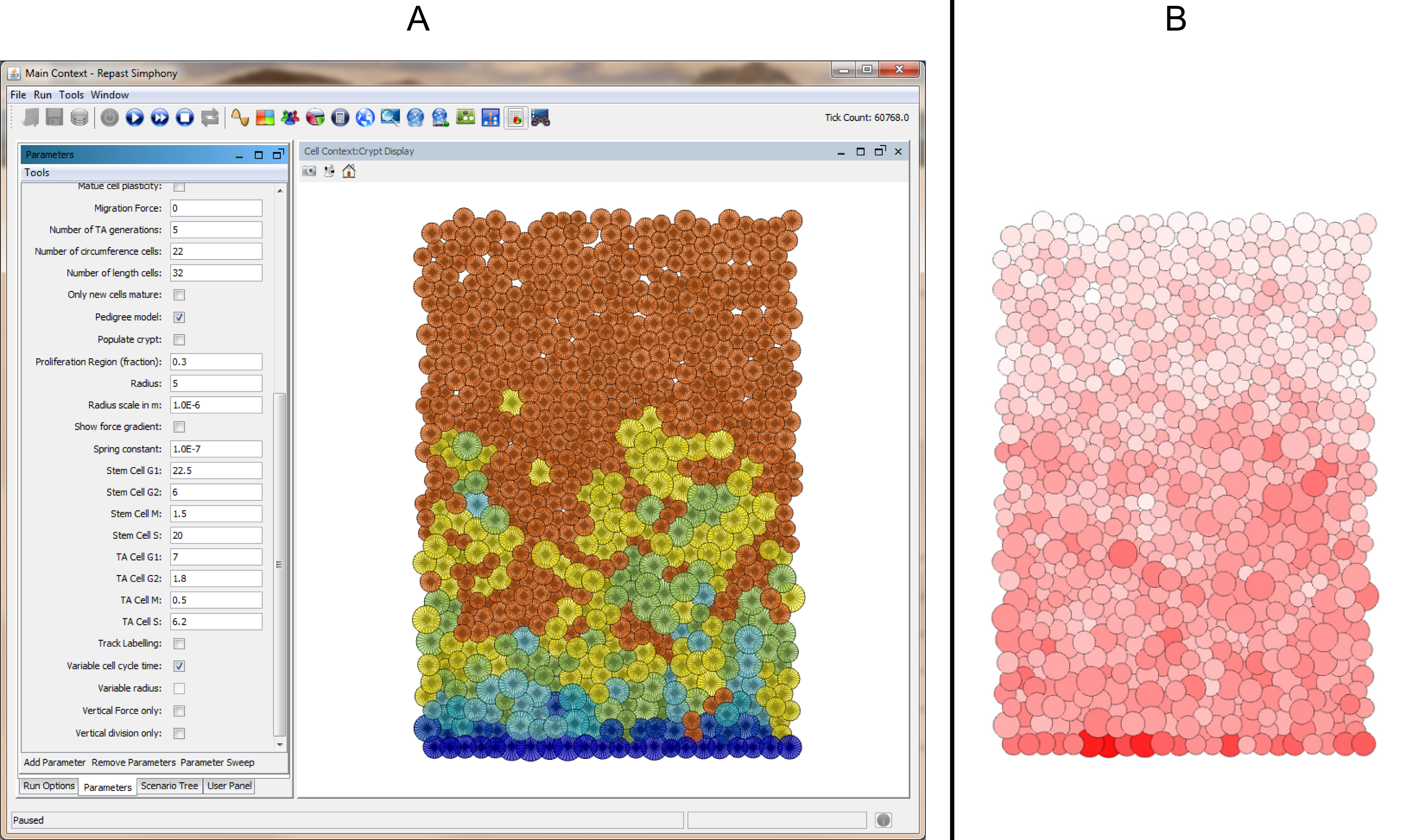

Supplement: Figure S3 — Our interactive modelling interface. A: Users have full control of model parameters, some of which can be changed during runtime (for example labelling cells in s-phase, or visualising the force gradient). B: A different view which shows the amount of force experienced by cells in the crypt. (TIFF) [file pone.0073204.s004.tiff]

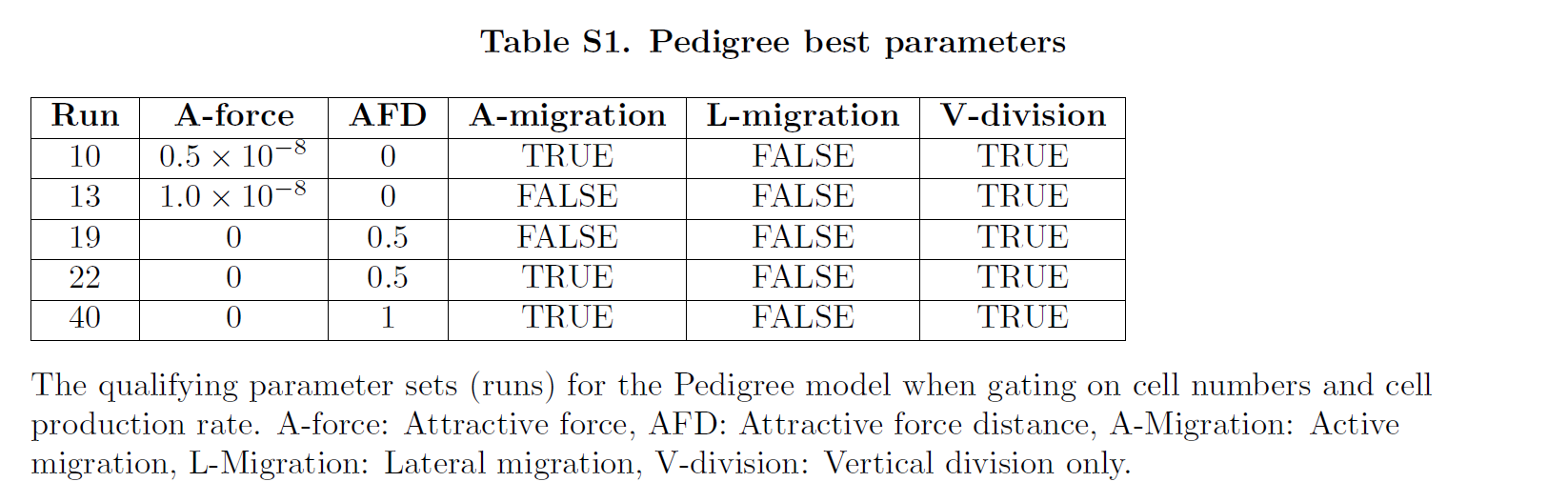

Supplement: Table S1 — Pedigree best parameters. The qualifying parameter sets (runs) for the Pedigree model when gating on cell numbers and cell production rate. A-force: Attractive force, AFD: Attractive force distance, A-Migration: Active migration, L-Migration: Lateral migration, V-division: Vertical division only. (TIFF) [file pone.0073204.s005.tiff]

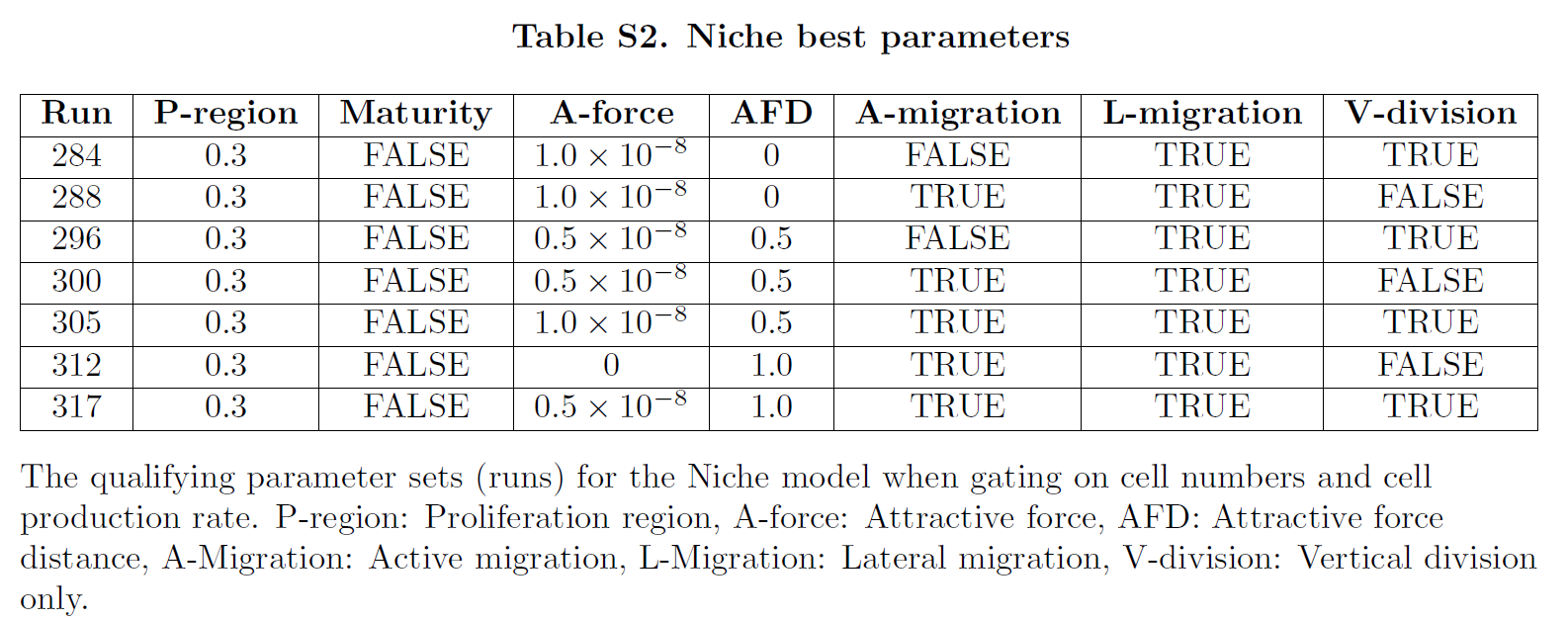

Supplement: Table S2 — Niche best parameters. The qualifying parameter sets (runs) for the Niche model when gating on cell numbers and cell production rate. P-region: Proliferation region, A-force: Attractive force, AFD: Attractive force distance, A-Migration: Active migration, L-Migration: Lateral migration, V-division: Vertical division only. (TIFF) [file pone.0073204.s006.tiff]
