# Supplementary material for: Cell Organisation in the Colonic Crypt: A Theoretical Comparison of the Pedigree and Niche Concepts
Source: PLoS One. 2013 Sep 12;8(9):e73204. doi: 10.1371/journal.pone.0073204 (PMC3771985; doi:10.1371/journal.pone.0073204)
Supplement: Text S1 — The system of ODE's that describe the pedigree model. (PDF) [file pone.0073204.s007.pdf]

Supporting Text S1
Cell organisation in the colonic crypt: a theoretical comparison of the pedigree and niche concepts.

Richard C. van der Wath[1,*], Bruce S. Gardiner[1], Antony W. Burgess[2,3], David W. Smith[1]
1 Engineering Computational Biology Group, School of Computer Science and Software Engineering, University of Western Australia, Perth, Australia
2 Ludwig Institute for Cancer Research, Royal Melbourne Hospital, Parkville, Victoria 3052 Australia
3 The Walter and Eliza Hall Institute of Medical Research, 1G Royal Parade, Parkville, Victoria 3052 Australia
∗ **E-mail: rvanderwath@gmail.com**

Ordinary Differential Equation Model

A very useful feature of the pedigree model is that it can be readily described by a system of linear Ordinary Differential Equations (ODEs). The rate of change in cell numbers for each cell population[1] is determined and when solved at steady state, gives an expression to estimate the stem cell division rate - an unknown quantity.

The ODE version of the pedigree model are given by the following equations:

$$
\begin{aligned}
S(t) &= s \\
\dot{T}_1(t) &= \alpha_S S - \alpha_{T_1} T_1 \\
\dot{T}_2(t) &= 2\alpha_{T_1} T_1 - \alpha_{T_2} T_2 \\
\dot{T}_3(t) &= 2\alpha_{T_2} T_2 - \alpha_{T_2} T_2 \\
&\vdots \\
\dot{T}_n(t) &= 2\alpha_{T_{n-1}} T_{n-1} - \alpha_{T_n} T_n \\
\dot{Z}(t) &= 2\alpha_{T_n} T_n - \gamma Z,
\end{aligned}
\qquad \text{(S1)}
$$

where $S(t) = s$ gives the constant number of stem cells, $T_i(t)$ gives the number of ith generation TA cells at time t, and $Z(t)$ gives the number of mature cells at time t. Cell division rates (average rate of the whole cell cycle) for stem cells and TA cells are given by α_S and α_{T_i} respectively.

[1] Cell population here refers to either stem cells, different TA cell generations, or mature cells.

The steady state solution of the system in S1 are given by

$$
\begin{aligned}
S^* &= s \\
T_1^* &= \frac{\alpha_S}{\alpha_{T_1}} S^* \\
T_2^* &= \frac{2\alpha_{T_1}}{\alpha_{T_2}} T_1^* \\
T_3^* &= \frac{2\alpha_{T_2}}{\alpha_{T_3}} T_2^* \\
&\vdots \\
T_n^* &= \frac{2\alpha_{T_{n-1}}}{\alpha_{T_n}} T_{n-1}^* \\
Z^* &= \frac{2\alpha_{T_n}}{\gamma} T_n^*.
\end{aligned}
\tag{S2}
$$

The expression for the steady state mature cell population can be simplified to $Z^* = 2^n \frac{\alpha_S}{\gamma} s$. Interestingly this expression is independent of the transit amplifying cell division rates (even when they are different between TA cell generations) but strongly depends on n the number of amplifying generations.

We can also derive an expression for the total number of cells at steady state, by simply adding up the individual steady state populations:

$$
\begin{aligned}
\text{Total} &= S^* + T_1^* + \cdots + T_n^* + Z^* \\
&= s + \frac{\alpha_S}{\alpha_{T_1}} S^* + \frac{2\alpha_{T_1}}{\alpha_{T_2}} T_1^* + \cdots + \frac{2\alpha_{T_n}}{\gamma} T_n^* \\
&= s + 2^0 \frac{\alpha_S}{\alpha_{T_1}} s + 2^1 \frac{\alpha_S}{\alpha_{T_2}} s + \cdots + 2^{n-1} \frac{\alpha_S}{\alpha_{T_n}} s + 2^n \frac{\alpha_S}{\gamma} s \\
&= s \left(1 + \alpha_S \left[2^0 \frac{1}{\alpha_{T_1}} + \cdots + 2^{n-1} \frac{1}{\alpha_{T_n}} + 2^n \frac{1}{\gamma} \right] \right).
\end{aligned}
\tag{S3}
$$

It is often assumed that TA cell generations have a homogeneous division rate, that is $\alpha_T \equiv \alpha_{T_1} = \alpha_{T_2} = \cdots = \alpha_{T_n}$. If this is the case the expression for the total cell population simplifies to

$$
\begin{aligned}
\text{Total} &= s \left(1 + \frac{\alpha_S}{\alpha_T} \left[2^0 + 2^1 + \cdots + 2^{n-1} \right] + \frac{\alpha_S}{\gamma} 2^n \right) \\
&= s \left(1 + \frac{\alpha_S}{\alpha_T} \left(2^n - 1 \right) + \frac{\alpha_S}{\gamma} 2^n \right).
\end{aligned}
\tag{S4}
$$

Reorganising gives us the expression for estimating α_S, the stem cell division rate:

$$
\alpha_S = \frac{\text{Total} \cdot \alpha_T \cdot \gamma - s \cdot \alpha_T \cdot \gamma}{s \cdot \gamma \cdot 2^n - s \cdot \gamma + s \cdot \alpha_T \cdot 2^n}.
\tag{S5}
$$
